# Comparative Transcriptome Analysis Reveals the Genes and Pathways Related to Wheat Root Hair Length

**DOI:** 10.3390/ijms25042069

**Published:** 2024-02-08

**Authors:** Jianbin Zeng, Yongmei Wang, Gang Wu, Qingyi Sun, Xiaoyan He, Xinyi Zhang, Xuelian Sun, Yan Zhao, Wenxing Liu, Dengan Xu, Xuehuan Dai, Wujun Ma

**Affiliations:** 1 College of Agronomy, Qingdao Agricultural University, Qingdao 266109, China; jianbin_zeng@qau.edu.cn (J.Z.); 20232101066@stu.qau.edu.cn (X.Z.); 202101034@qau.edu.cn (X.D.); 2 Academy of Dongying Efficient Agricultural Technology and Industry on Saline and Alkaline Land in Collaboration with Qingdao Agricultural University, Dongying 257347, China

**Keywords:** wheat, root hair length, transcriptome sequencing, differentially expressed genes (DEGs), pathway analysis

## Abstract

Tube-like outgrowths from root epidermal cells, known as root hairs, enhance water and nutrient absorption, facilitate microbial interactions, and contribute to plant anchorage by expanding the root surface area. Genetically regulated and strongly influenced by environmental conditions, longer root hairs generally enhance water and nutrient absorption, correlating with increased stress resistance. Wheat, a globally predominant crop pivotal for human nutrition, necessitates the identification of long root hair genotypes and their regulatory genes to enhance nutrient capture and yield potential. This study focused on 261 wheat samples of diverse genotypes during germination, revealing noticeable disparities in the length of the root hair among the genotypes. Notably, two long root hair genotypes (W106 and W136) and two short root hair genotypes (W90 and W100) were identified. Transcriptome sequencing resulted in the development of 12 root cDNA libraries, unveiling 1180 shared differentially expressed genes (DEGs). Further analyses, including GO function annotation, KEGG enrichment, MapMan metabolic pathway analysis, and protein–protein interaction (PPI) network prediction, underscored the upregulation of root hair length regulatory genes in the long root hair genotypes. These included genes are associated with GA and BA hormone signaling pathways, FRS/FRF and bHLH transcription factors, phenylpropanoid, lignin, lignan secondary metabolic pathways, the peroxidase gene for maintaining ROS steady state, and the ankyrin gene with diverse biological functions. This study contributes valuable insights into modulating the length of wheat root hair and identifies candidate genes for the genetic improvement of wheat root traits.

## 1. Introduction

Tube-like extensions emerge from specialized epidermal cells, forming root hairs that significantly enhance root surface area and improve water and nutrient absorption [1]. The lack of root hairs in *Arabidopsis* has been linked to reduced water absorption and decreased drought tolerance [2]. Barley studies have demonstrated that root hairs are essential for absorbing water, especially in rapidly transpiring plants facing drying soils, as evidenced by analyses of xylem suction and transpiration rates in both wild-type and hairless mutant plants [3]. Mutant plants possessing impaired growth of root hairs exhibit deficiencies in the process of ion nutrient uptake and the production of biomass, with the accumulation of nutrients positively correlated with the length of root hairs under conditions of nutrient deficiency [4,5]. Genotypes of barley and wheat with longer root hairs demonstrate better adaptation to low-nutrient soils [6,7].

Root hairs are essential for enhancing seedling survival during soil disruption by facilitating root anchoring and providing traction for root tip penetration into the soil [8,9]. As the root system matures, the growth of root hairs and the secretion of adhesive molecules contribute to the formation of rhizosheaths [10], offering plants a means of adaptation to diverse biotic and abiotic conditions. Rhizosheaths serve as protective barriers against the loss of water, offer plant parasites and mechanical defense against herbivores, and help in obtaining nutrients and water [11]. The relationship between rhizosheath weight and root hair length in wheat has been found to be substantially correlated [12]. Critical ion transport systems, such as the Shaker channels AtAKT1 and AtKC1 and K^+^ transporter AtHAK5 [13,14], as well as NO_3_^−^ transporters NPF, NRT2, and NRT3 [15,16], are located on the root hair’s plasma membranes and play indispensable roles in potassium and nitrate absorption. Additionally, root hairs in *Arabidopsis* contain members of the Sultr, PHT1, and AMT families which enable the uptake of ammonium [17], phosphate [18], and sulfate [19], respectively. Plants release numerous organic compounds in the soil, creating a nutrient-rich rhizosphere conducive to microbial community development. Plant growth-promoting rhizobacteria (PGPR) generate beneficial interactions with the roots and promote the growth of plants through various mechanisms. These include the provision of protection against phytoparasites, production of phytohormones influencing root development, and the solubilization of poorly soluble nutrient sources [20]. Comparative studies on wild-type and hairless mutant barley plants revealed a threefold higher carbon exudation in wild-type plants [21]. Furthermore, experiments with both wild-type and hairless mutant barley plants demonstrated that a lack of root hairs led to a substantial decrease in the diversity of the bacterial community [22]. It has been suggested that root hairs serve as a means of expelling heavy metals, including cadmium and lead, conferring greater resistance to these substances [23]. The symbiotic interaction between legume root hairs and rhizobial bacteria forms a novel root structure called the nodule. The onset of the interaction between rhizobial bacteria and legumes is indicated by observable modifications in root hair morphology, including branching, swelling, and curling [24].

The growth and development of root hairs can be broadly categorized into four stages, including maturation, apical growth, initiation, and root hair cell fate determination [25]. Environmental factors, such as nutrient availability, the presence of a microbial community in the rhizosphere, soil porosity, strength, and water content, significantly affect the length and abundance of root hairs [26]. Phytohormones, including ethylene, cytokinin, jasmonic acid, auxin, and strigolactone, have been found to stimulate root hair growth, whereas abscisic acid and brassinosteroids act as inhibitors [25]. The growth of root hairs is significantly affected by the availability of nutrients in the soil, particularly inorganic phosphate [27]. *Arabidopsis* mutants that lack regularly shaped root hairs under a sufficient provision of phosphate generate normally shaped root hairs under phosphate deficiency [28]. Root hair development and growth are crucial in arid circumstances, as indicated in previous research [29,30]. Conversely, both root hair density and length decrease under salt stress [31,32]. The development and expansion of root hairs are closely linked to changes in the pH levels of the apoplast and cytosol, suggesting dynamic alterations in the activity of H^+^-ATPase within the plasma membrane [33]. Furthermore, the normal development of root hairs requires the reactive oxygen species (ROS) accumulated at the tip of root hair [34].

As suggested, yield stability can be improved by the selection of advantageous root hair characteristics without compromising yield potential, addressing the challenge of enhancing both productivity and resilience. Preserving or enhancing root hairs emerges as a crucial trait in breeding the next generation of crops to optimize water and nutrient absorption [1,35,36]. Wheat (*Triticum aestivum* L.) is a widely cultivated cereal plant in diverse global growing environments. Consequently, the exploration of wheat varieties characterized by well-developed root hairs and the investigation of genes responsible for the development of root hair hold significant importance in cultivating stress-resistant and high-yielding wheat varieties.

The tip growth, initiation, and elongation of root hairs are governed by diverse yet interconnected molecular pathways [37]. Thus, it is crucial to enhance our understanding of the root hair development pathway. Next-generation sequencing (NGS) platforms have enabled the comprehensive exploration of the complexity and regulation of gene expression networks in various crop species [38]. Transcriptome studies utilizing contrasting lines have been conducted on several crops, such as barley, to unravel the root hair gene regulatory network [39]. However, there is a scarcity of transcriptomic research on root hairs using contrasting wheat genotypes.

In this study, 2 long root hair genotypes and 2 short root hair genotypes were identified from 261 different wheat genotypes based on root hair length. Through transcriptome sequencing, a set of genes related to root hair growth, including those involved in amino acid synthesis, hormone response, secondary metabolism, anchor proteins, and antioxidant genes, were identified. This study contributes to the enrichment of the theory of wheat root hair growth regulation and provides candidate genes for genetic improvement in wheat.

## 2. Results

### 2.1. Measurement of Wheat Root Hair Length and Screening of Long Root Hair Genotypes

**Figure 1:**
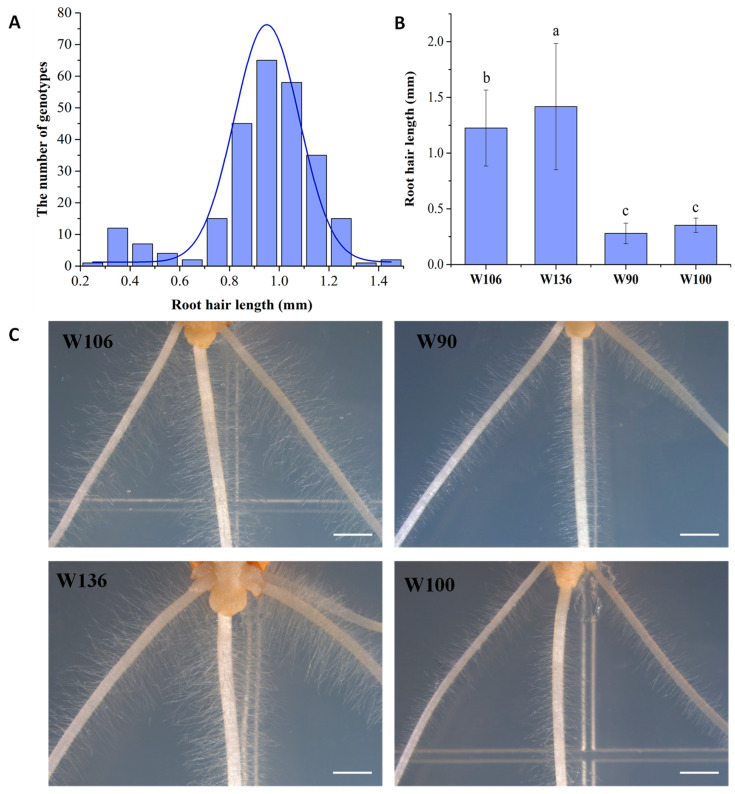
Statistical analysis of root hair length and screening of extreme materials for 261 wheat samples of different genotypes. (**A**) Frequency distribution of root hair length across 261 wheat samples. (**B**) Histogram depicting root hair length distribution in four extreme materials. Different letters represent significant differences (*p* < 5%). (**C**) Visual representation of the root hair phenotypes of the four extreme materials. W106 and W136 are long root hair genotypes. W90 and W100 are short root hair genotypes. Bar = 1.05 mm.

The root hair length of 261 wheat samples from various genotypes of taproots cultured for 4 d was measured using the agar culture dish method. The root hair length distribution across the different wheat genotypes displayed a predominantly normal distribution with a slight right deviation (Figure 1A). Significant variations in root hair length were observed among wheat samples of different genotypes, with the maximum, minimum, mean, standard deviation (SD), and coefficient of variation values for root hair length being 1.43 mm, 0.28 mm, 0.98 mm, 0.21 mm, and 22.34, respectively (Supplemental Table S1). Based on the root hair length measurements, two long root hair genotypes (W106 and W136), with root hair lengths of 1.23 mm and 1.42 mm, and two short root hair genotypes (W90 and W100), with root hair lengths of 0.28 mm and 0.35 mm, were selected. A substantial root hair length difference was observed between the long and short root hair genotypes (Figure 1B). Stereomicroscopic observations further confirmed that the root hair lengths of W106 and W136 were significantly larger than those of W90 and W100 (Figure 1C).

### 2.2. RNA-Sequencing Data Output and Screening of DEGs

To uncover the regulatory genes associated with wheat root hair growth, root transcriptome sequencing was performed on two long root hair genotypes (W106 and W136) and two short root hair genotypes (W90 and W100) using the Illumina platform. The sequencing results from the 12 libraries revealed clean read counts ranging from 46,352,918 to 97,690,332. The percentages of clean reads aligned to the reference genome were 90.04–94.19%, with 82.57–87.55% being uniquely mapped clean reads and 6.64–7.5% being multiple mapped clean reads. Each sample contained more than 80,000 genes (Table 1), providing ample data for further DEG analysis.

**Figure 2:**
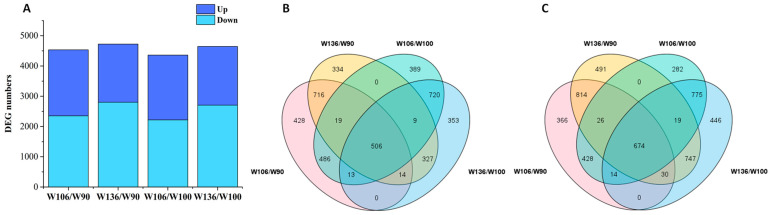
DEGs in long root genotypes (W106 and W136) and short root genotypes (W90 and W100). (**A**) Number of DEGs identified in long root genotypes compared to short root genotypes. (**B**) Shared upregulated genes in long and short root genotypes. (**C**) Shared downregulated genes in the long and short root genotypes. DEGs were selected based on |log2^Fold Change^| ≥ 2 and FDR < 0.05, where fold changes were W106/W90, W106/W100, W136/W90, and W136/W100, respectively.

Differential gene expression between wheat samples with long and short root hair genotypes was assessed using Fragments Per Kilobase of transcript per million fragments mapped (FPKM) values. Genes meeting the criteria of |log_2_^Fold Change^| ≥ 2 and FDR < 0.05 were considered as DEGs, where fold change values represented W106/W90, W106/W100, W136/W90, and W136/W100, respectively. DEG analysis revealed that W106/W90 had 4534 DEGs (2182 upregulated and 2352 downregulated), W106/W100 had 4360 DEGs (2142 upregulated and 2218 downregulated), W136/W90 had 4726 DEGs (1925 upregulated and 2801 downregulated), and W136/W100 had 4648 DEGs (1942 upregulated and 2706 downregulated) (Figure 2A). Across the four genotypes, 1180 shared DEGs were identified (506 upregulated (Figure 2B) and 674 downregulated (Figure 2C)) (Supplemental Table S2). These shared DEGs in the long root hair genotypes/short root hair genotypes were considered as genes associated with wheat root hair growth.

### 2.3. Validation of DEGs Expression Levels

**Figure 3:**
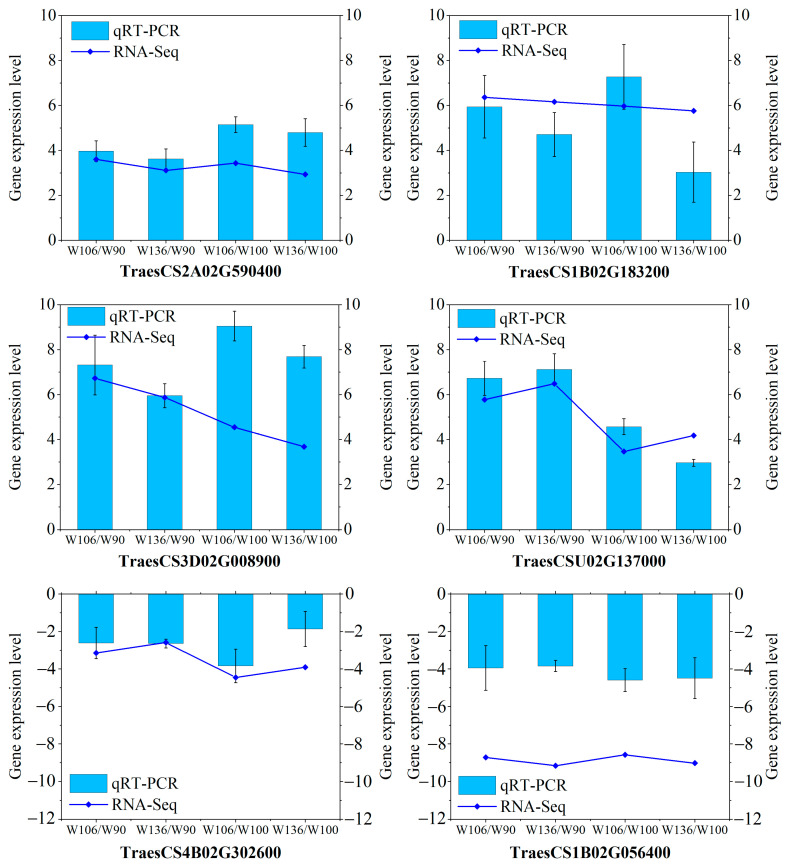
qRT-PCR was employed to confirm the expression levels of DEGs. The bar graph represents qRT-PCR results, with the relative expression of genes calculated using the −ΔΔCt method. The fold-line graph depicts the RNA-Seq results, expressing the values as a log2^Fold Change^. The fold changes were W106/W90, W106/W100, W136/W90, and W136/W100.

To validate the transcriptome sequencing results, six randomly selected genes (TraesCS1B02G056400 (ankyrin like gene), TraesCS4B02G302600 (transcription factor MADS-box-like gene), TraesCS1B02G183200 (myosin-related gene), TraesCS3D02G008900 (transcription factor bHLH-like gene), TraesCS2A02G590400 (sugar transporter gene), and TraesCSU02G137000 (peroxidase-like gene)) were subjected to qRT-PCR. qRT-PCR expression patterns were highly consistent with the RNA-Seq results (Figure 3), confirming the reliability and accuracy of the RNA-Seq data. Specifically, the expression levels of TraesCS1B02G056400 and TraesCS4B02G302600 in the long root hair genotypes were lower than those in the short root hair genotypes. Conversely, the other four genes in the long root hair genotypes were higher than those in the short root hair genotypes.

### 2.4. GO (Gene Ontology) Annotation and Enrichment Analysis of the Shared DEGs in Four Genotypes

**Figure 4:**
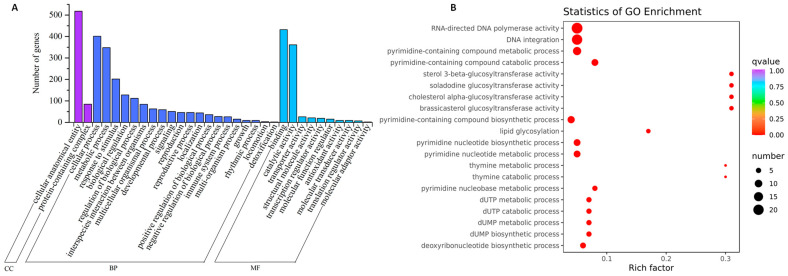
GO function annotation classification and enrichment of 1180 shared DEGs. (**A**) GO classification of the shared DEGs, where CC denotes cellular component (purple column), BP represents biological process (dark blue column), and MF stands for molecular function (light blue column). (**B**) GO enrichment of shared DEGs. The vertical axis illustrates GO-enriched entries, whereas the horizontal axis represents the Rich Factor, indicating the ratio of DEGs annotated to the entry to the total number of genes annotated to the entry. Larger Rich Factor values indicate greater enrichment. The dot size corresponds to the number of genes enriched in the pathway, with larger dots indicating more genes, and smaller dots indicating fewer genes. The dot color reflects the significance of pathway enrichment, as assessed by q value. A redder color closer to 0 indicates more significant enrichment.

The GO function annotation classification of the 1180 shared DEGs revealed categorization into the cellular component, biological process, and molecular function, encompassing 2, 20, and 10 secondary GO term categories, respectively (Figure 4A). Notably, DEGs annotated to the cellular anatomical entity (GO:0110165) constituted 86% of the cellular component category. In the biological process category, response to stimulus (GO:0050896), metabolic process (GO:0008152), and cellular process (GO:0009987) were the predominant GO term categories, with DEGs accounting for 12%, 20%, and 23%, respectively. In the molecular function category, catalytic activity (GO:0003824) and binding (GO:0005488) were the primary GO term categories, with DEGs representing 40% and 48%, respectively.

The GO enrichment analysis of DEGs indicated that among the top 20 significantly enriched GO terms, 5 were related to molecular function and 15 were associated with the biological process category (Figure 4B). Notably, RNA-directed DNA polymerase activity (GO:0003964) was the most significantly enriched in the molecular function category, with a ratio of 22 DEGs annotated to this GO term out of a total of 459 background genes annotated to this term. Within the molecular function category, the remaining four enriched GO terms (GO:0016906, GO:0102202, GO:0102203, and GO:0102205) were functionally correlated with glucosyltransferase activity. In the domain of biological processes, the GO term DNA integration (GO:0015074) exhibited the most pronounced enrichment, with 20 DEGs annotated to this term out of a total of 420 background genes. Among the remaining biological process GO terms, except for GO:0030259, which was involved in the lipid glycosylation route, the remaining 13 GO terms (GO:0072527, GO:0072529, GO:0072528, GO:0006221, GO:0006220, GO:0006210, GO:0019859, GO:0006206, GO:0009263, GO:0006226, GO:0046078, GO:0046080, and GO:0046081) were all associated with pyrimidine nucleotide metabolism.

### 2.5. KEGG Classification and Enrichment Analysis of the Shared DEGs in Four Genotypes

**Figure 5:**
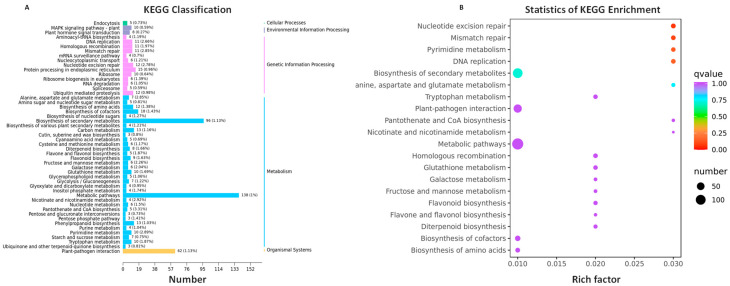
KEGG classification and enrichment of 1180 shared DEGs. (**A**) KEGG classification of shared DEGs. The horizontal axis indicates the number of DEGs annotated to the pathway, and the vertical axis indicates the name of the KEGG pathway. The ratio of the DEGs annotated to the pathway to the number of background genes annotated to the pathway is shown in parentheses. The rightmost label represents the classification to which the KEGG pathway belongs. (**B**) KEGG enrichment of shared DEGs. The vertical axis denotes the KEGG enrichment entry, and the horizontal axis denotes the Rich Factor, indicating the ratio of DEGs annotated to the entry to the total number of genes annotated to the entry. Larger Rich Factor values indicate greater enrichment. The dot size represents the number of genes enriched in the pathway, with larger dots indicating more genes, and smaller dots indicating fewer genes. The dot color represents the significance of pathway enrichment, as assessed by q value. A redder color closer to 0 indicates more significant enrichment.

KEGG classification revealed that the top 50 KEGGs, with the highest number of annotated DEGs, could be classified into five groups: organismal systems, metabolism, genetic information processing, environmental information processing, and cellular processes, with 1, 33, 13, 2, and 1 KEGG pathways, respectively (Figure 5A). Among these pathways, other terpenoid-quinone biosynthesis (ko00130), ubiquinone, pentose phosphate pathway (ko00030), pentose and glucuronate interconversions (ko00040), and wax, suberine, and cutin biosynthesis (ko00073) had the lowest number of annotated DEGs, each with only three. Conversely, the metabolic pathway (ko01100) had the highest number of annotated DEGs, totaling 138 (Figure 5A).

KEGG enrichment analysis identified the top 20 significantly enriched KEGGs, which could be classified into organismal systems, metabolism, and genetic information processing comprising 1, 15, and 4 KEGG pathways, respectively (Figure 5B). Notably, DNA replication (ko03030), pyrimidine metabolism (ko00240), nucleotide excision repair (ko03420), and mismatch repair (ko03430) exhibited greater enrichment significance compared to other KEGG pathways. The Rich Factor values, representing the proportion of DEGs within the KEGG pathway relative to the amount of genes annotated in the KEGG pathway, were 2.66% (11 out of 413), 2.89% (10 out of 346), 2.78% (12 out of 431), and 2.85% (11 out of 386), respectively (Figure 5A,B).

### 2.6. Pathway Analysis of the Shared DEGs in Four Genotypes Using MapMan

**Figure 6:**
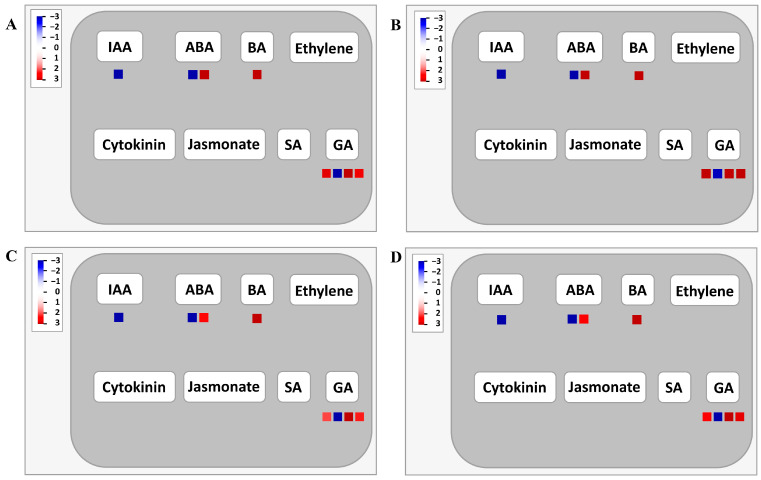
Expression analysis of specific DEGs related to root hair growth in hormone pathways using MapMan visualization tool. (**A**) W106/W90, (**B**) W136/W90, (**C**) W106/W100, (**D**) W136/W100. Upregulated genes are represented in red, and downregulated genes are depicted in blue. IAA: indoleacetic acid, ABA: abscisic acid, BA: benzylaminopurine, SA: salicylic acid, GA: gibberellin.

**Figure 7:**
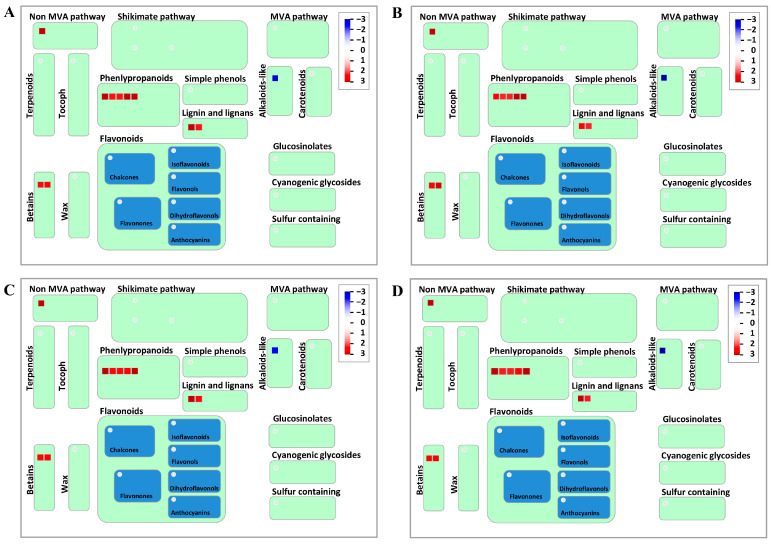
Expression analysis of specific DEGs involved in root hair growth in secondary metabolic pathways using MapMan visualization tool. (**A**) W106/W90, (**B**) W136/W90, (**C**) W106/W100, (**D**) W136/W100. Upregulated genes are represented in red, and downregulated genes are depicted in blue. MVA pathway: mevalonate pathway, Non-MVA pathway: non-mevalonate pathway.

To gain deeper insights into the functional classification of the 1180 shared DEGs, an analysis of the metabolic pathways involving these DEGs was conducted using MapMan v 3.6.0RC1. The results revealed that 329 DEGs were distributed across 28 different pathways among 35 pathway types (Supplemental Table S3). Among these pathways, the DNA damage response, plant reproduction, and clade-specific metabolism exhibited the lowest number of DEGs, each containing only one DEG. Conversely, RNA biosynthesis emerged as the pathway with the highest number of DEGs (21 DEGs). Solute transport and protein modification followed closely, with 19 and 18 DEGs, respectively. Other pathways were comprised of 3–15 DEGs (Supplemental Table S3). In the regulation overview pathway, hormone metabolism featured prominently, with 8 DEGs (5 upregulated and 3 downregulated). Notably, among the four DEGs involved in gibberellin (GA) metabolism, traescs5B02G302100, traescs2B02G081600, and traescs5B02G013600 exhibited upregulation (Figure 6). Additionally, the secondary metabolism pathway identified nine DEGs, with traescs5A02G115200 being the only downregulated gene. The remaining seven genes (traescs2A02G175200, traescs2A02G592500, traescs1B02G425300, traescs2A02G560400, traescs5A02G358600, traescs7B02G391100, and traescs7D02G276800) were all upregulated. Notably, the traescs2A02G560400 and traescs5A02G358600 genes played a role in the phenylpropanoid pathway and the lignin and lignan pathways (Figure 7).

### 2.7. PPI Network of Shared DEGs Encoding Proteins

**Figure 8:**
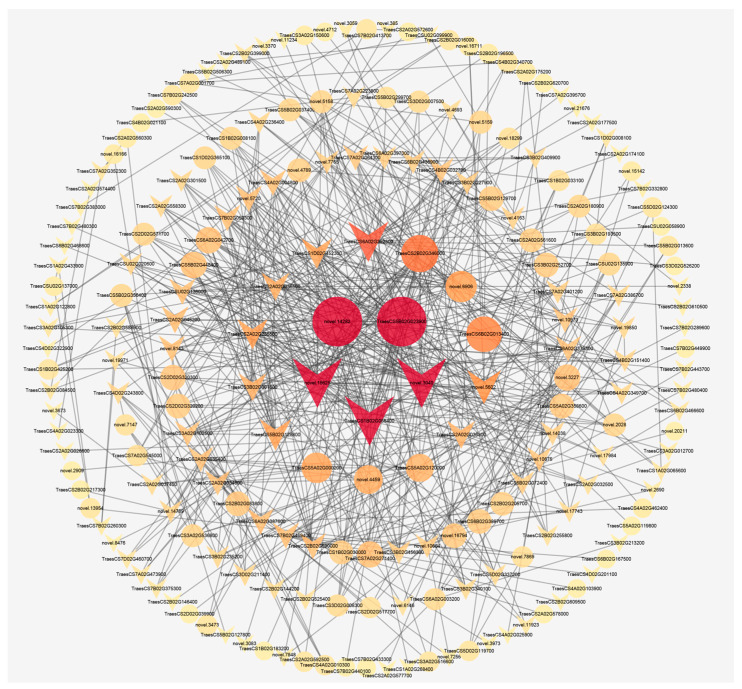
Protein–protein interactions of shared DEGs in W106/W90, W136/W90, W106/W100, and W136/W100. Circles represent upregulated genes, and triangles represent downregulated genes. Larger circles and triangles with redder colors indicate genes with more interacting proteins.

PPIs among the proteins encoded by the 1180 shared DEGs were predicted using wheat annotation information from the String Database. We identified 1301 interaction pairs, with 609 pairs having a combined score greater than 0.6 (Figure 8). Among the centrally located genes, TraesCS5B02G023900 (upregulated), novel.14282 (upregulated), TraesCS1B02G056400 (downregulated), novel.3040 (downregulated), and novel.16625 (downregulated) were annotated as ankyrin-3-like, ankyrin repeat and SOCS box protein 7-like isoform X1, ankyrin-3-like isoform X2, hypothetical protein CFC21_028432, and unnamed protein, respectively, in the non-redundant protein sequence database. Interactions were observed between these five genes, with 34 interaction pairs involving other DEGs. In the second circle of genes from inside to outside, six upregulated genes, TraesCS2B02G346000 (hypothetical protein CFC21_024063), TraesCS6B02G013400 (hypothetical protein CFC21_085837), novel.6906 (uncharacterized protein LOC125544633 isoform X2), TraesCS5A02G000200 (NARROW LEAF 1-like), novel.4459 (hypothetical protein B296_00032057), and TraesCS5A02G120000 (2-oxoisovalerate dehydrogenase subunit alpha 2, mitochondrial-like), displayed 23, 21, 16, 16, 13, and 12 interaction pairs with other DEGs, respectively. Similarly, eight downregulated genes, TraesCS6A02G392500 (unnamed protein), novel.5602 (uncharacterized protein LOC109758199), TraesCS5B02G128800 (hypothetical protein CFC21_070821), TraesCS1D02G452300 (uncharacterized protein LOC109754656), TraesCS2A02G235500 (ADP-ribosylation factor 1), TraesCS3B02G001600 (uncharacterized protein LOC123068142), TraesCS2A02G036500 (UMP-CMP kinase 2-like isoform X1), and TraesCS2A02G036300 (acetyl-CoA acetyltransferase, cytosolic 1-like), exhibited 27, 19, 16, 15, 15, 12, 12, and 12 interaction pairs with other DEGs, respectively. The remaining DEGs showed fewer than 10 interaction pairs, with 48 DEGs having only 1 interaction pair.

## 3. Discussion

### 3.1. Root Hairs Play a Key Role in Water and Nutrient Uptake by Plants

**Figure 9:**
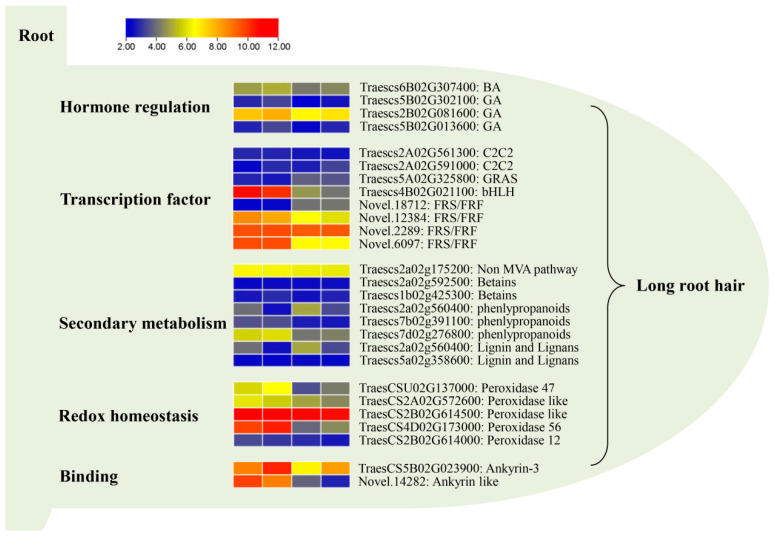
Key DEGs regulating the long root hair phenotype of W106 and W136 (from left to right: W106/W90, W136/W90, W106/W100, and W136/W100). The redder the color, the greater the upward multiplier.

The root system serves as a vital organ for normal plant life activities, primarily facilitating water and nutrient absorption. To enhance these functions, plants strategically increase their surface area through mechanisms such as root hair expansion [40]. Root hairs, unicellular tubular extensions of root epidermal cells, play a crucial role in water and nutrient absorption as well as in abiotic stress resistance [1,41]. Under nutrient-deficient conditions, plants exhibit induced root hair development. For instance, in phosphorus-deficient soils, plants augment root hair density to improve phosphorus absorption [42]. Similar responses were observed under conditions of low nitrogen and potassium [43,44]. Additionally, the growth of root hairs increases water absorption capacity and enhances drought resistance [30,39]. Therefore, the regulation of root hair growth under drought stress is a focal point for understanding plant drought tolerance mechanisms [45]. Although previous studies on wheat roots have primarily focused on development, physiological functions, and stress tolerance (e.g., drought resistance and salt tolerance) [46], the molecular mechanisms underlying wheat root hair development have remained unclear. This study addresses this gap by analyzing the root hair length of 261 wheat samples and screening two long root hair genotypes (W106 and W136) and two short root hair genotypes (W90 and W100). Subsequent transcriptome sequencing of the selected materials revealed the functions of DEGs and the associated metabolic pathways. Several identified genes involved in hormone signaling pathways, transcriptional regulation, secondary metabolic pathways, redox steady state, and stress tolerance contributed to the regulation of wheat root hair length (Figure 9). This study significantly enriches our understanding of the regulatory mechanisms governing wheat root hair length and provides valuable genetic resources for enhancing both yield and stress tolerance in wheat.

### 3.2. GA and BA Are Key Factors Regulating Root Hair Elongation

In addition to nutrient stress and drought, hormones play a crucial role in signaling for root hair growth regulation. Gibberellins (GAs) are known regulators of cell elongation, root meristem growth, and lateral root formation [47]. Although direct evidence on GA’s role in root hair growth regulation is limited, Devlin and Brown’s study [48] demonstrated that GA positively influences root hair elongation in *Agrostis alba*. Exogenous GA application has been shown to increase the number and length of *Datura innoxia* root hairs [49]. Another study by Zhang et al. [50] explored the effects of auxin (indole-3-acetic acid, IAA), ethylene biosynthesis precursor (1-aminocyclopropane-1-carboxylic acid, ACC), and cytokinin (6-benzylaminopurine, BA) on *Arabidopsis* root hair growth. ACC plays a unique role in the initial phase of root hair elongation, whereas IAA, ACC, and BA collectively promote root hair elongation in the subsequent stages, with BA acting independently of IAA and ACC. Exogenous BA application restores root hair elongation in the absence of IAA and ACC. In this study, analysis of the hormone signaling pathway identified one BA and two GA pathway genes. Their expression levels in the long root hair genotypes exceeded those in the short root hair genotypes (Figure 6), indicating their involvement in regulating wheat root hair elongation.

### 3.3. Transcription Factors Such as FRS/FRF and bHLH Are One of the Influencing Factors of Root Hair Length

The FAR1-RELATED SEQUENCE (FRS)/FRS-RELATED FACTOR (FRF) family, a pivotal class of light-signaling proteins, participates in far-red light signal transduction. Evolved from the ancient Mutator transposase, FRS/FRF acts as a transcription factor capable of specifically recognizing the *cis*-regulatory element, FBS. It regulates the transcription levels of multiple downstream target genes, influencing essential life processes, such as plant light signaling, growth, development, and stress response [51]. Genetic evidence highlights the role of FHY3, a significant FRS/FRF family member, in promoting aboveground branching and maintaining aboveground tissue function [52,53]. However, their impact on root hair growth remains unexplored. The basic helix-loop-helix (bHLH) transcription factor, a substantial plant family, plays a crucial role in diverse life processes. In *Arabidopsis*, five bHLH genes (RHD6, RSL1, RSL2, LRL1, and LRL2) are inhibited by GLABRA2 (GL2) in non-hair cells (N cells), whereas in hair cells (H cells), the absence of GL2 activation allows for the normal expression of these bHLH genes, promoting root hair growth and development [54]. Loss of function of another bHLH transcription factor, RSL4, leads to the short root hair phenotype in *Arabidopsis*, whereas constitutive RSL4 expression maintains long root hair features [55]. The GRAS transcription factor, recognized for its multifunctionality, actively participates in diverse biological processes, such as plant development and growth, metabolism, and responses to both abiotic and biotic stressors [56]. The highly expressed transcription factors identified in this study, exclusive to long root hair genotypes, included FRS/FRF, bHLH, and GRAS (Figure 9). This suggests their roles in the growth and development of root hair.

### 3.4. Secondary Metabolic Pathway Is an Influencing Factor of Root Hair Length

The phenylpropanoid pathway, a pivotal route for synthesizing metabolites such as lignin, flavonoids, tannins, and anthocyanins, is under the regulation of the MYB transcription factor, significantly impacting plant growth and development [57]. Previous studies have established a role for *GbMYBR1* in *Arabidopsis* phenylpropanoid synthesis and epidermal hair development [58]. Buckwheat hairy roots exhibit a heightened expression of genes associated with the phenylpropanoid pathway [59]. Moreover, environmental stresses including low nitrogen, high temperature, and drought can stimulate the activation of the phenylpropanoid pathway [60,61]. Our study identified phenylpropanoid pathway genes with an elevated expression in long root hair genotypes compared to short root hair genotypes, suggesting their potential involvement in the development and growth of root hair. Lignin and lignan, integral components of the cell wall in vascular plants, play vital roles in providing structural support and transport mechanisms [62]. They also serve as barriers against soil-borne pathogens and influence the inter-root microbial community [63]. Proteomic analyses of root hair cells imply that some proteins related to lignin and lignan metabolism contribute positively to rapid cell wall elongation and overall root hair elongation [64,65]. In this study, two genes associated with lignin and lignan synthesis exhibited higher expression in the long root hair genotypes (Figure 7), indicating a potential connection to root hair elongation.

### 3.5. Peroxidase Plays a Key Role in the Regulation of Root Hair Growth

The anomalous accumulation of reactive oxygen species (ROS) in root hair cells can detrimentally affect cellular growth, potentially leading to cell rupture. For example, the introduction of exogenous H_2_O_2_ impedes the polar expansion of root hair, whereas the application of ROS scavengers reduces cell rupture. Hence, maintaining a steady state of ROS proves important in regulating cell elongation by preserving the cell wall properties [66,67]. Apoplast class III peroxidase (PRX) is a key regulator in sustaining ROS equilibrium and serves as a multifunctional factor for rapid cell elongation [68]. In a study by Marzol et al. [69], the investigation of PRXs (PRX73, PRX44, and PRX01) revealed their roles in mediating the root hair growth regulation through interactions with cell wall extensins (EXTs). This study demonstrated that the PRX44 and PRX73 overexpression and *prx01*, *44*, *73* triple mutation strains exhibited contrasting performance in ROS accumulation, peroxidase activity, root hair growth, and significantly influenced cell wall thickness. This underscores the pivotal role of PRX01, PRX44, and PRX73 in the polar elongation of *Arabidopsis* root hair cells through the regulation of cell wall properties via interaction with EXT. Pacheco et al. [70] reported that PRX62 and PRX69 serve as key genes in *Arabidopsis* root hair growth. These genes play crucial roles in maintaining ROS equilibrium and regulating EXT-induced cell wall stability, particularly under low-temperature conditions. The highly expressed antioxidant enzymes identified in this study, primarily peroxidase (Figure 9), suggest their significance in regulating cellular ROS equilibrium and in facilitating root hair cell elongation.

### 3.6. Ankyrin May Be a Key Factor Regulating Wheat Root Hair Growth

The gene family of Ankyrin (ANK) serves as a member of the largest gene families in plants. Within this family, ANK repeat sequences function as scaffolds for PPI and play crucial roles in diverse cytological and biological processes. These processes encompass intercellular signal transduction, maintenance of cytoskeletal integrity, transcription, cell cycle regulation, and stress responses [71,72,73]. Proteins featuring ANK repeated sequences have demonstrated involvement in vital biological processes in plants, such as lateral root development [74], pollen germination and pollen tube growth [75], chloroplast-associated protein transport [76], and overall plant growth and development [77]. Furthermore, these proteins are key players in response to both biotic stress (e.g., fungi, bacteria, and viruses) [73,78,79] and abiotic stress (e.g., drought, salt, and high and low temperature) [80,81,82], in addition to influencing plant hormone signaling pathways (e.g., abscisic acid, ethylene, and salicylic acid) [83,84,85]. A case in point is the TIP GROWTH DEFECTIVE1 (TIP1) gene in *Arabidopsis*, which encodes a protein containing ANK repeated sequences. Mutants of TIP1 exhibit significantly shorter root hair length than wild-type plants [86]. In this study, the identification of two highly expressed ANK genes in long root hair genotypes, centrally positioned in the PPI network (Figure 8), underscores their importance as regulators of wheat root hair elongation.

## 4. Materials and Methods

### 4.1. Plant Materials

A sample of 261 wheat genotypes, comprising both old and modern varieties, underwent characterization for root hair length measurements. Detailed information, including name and root hair length, is provided in Supplemental Table S4.

### 4.2. Observation and Length Measurements of Root Hairs

The sterilization of wheat seeds was performed with 2% H_2_O_2_ for 30 min, they were washed five times with distilled water, and germinated in moistened filter paper-covered germination boxes. Whitish seeds were then transferred to an agar medium for further incubation (22 °C/18 °C, 16 h/8 h). On the fourth day of germination, root hair growth was observed, and taproot photographs were captured using a stereomicroscope (Leica TL 5000 Ergo Transmitted Light Base, Heerbrugg, Switzerland). Root hair phenotypes were observed following the description by He et al. [39] with some modifications. For each genotype, six biological replicates were conducted, and one taproot photograph was taken for each replicate. Root hair length was measured using Image-Pro Plus 6.0. Four identical parts of the taproot were selected from all photographs to measure the root hair length, resulting in 24 values for each genotype. Root hair length for each genotype was determined as the average of these 24 values, and comparisons among wheat samples of different genotypes were made based on the mean value.

### 4.3. Sample Preparation and RNA Extraction

Two long root hair genotypes (W106 (Jingyang60) and W136 (Honggoudou)) and two short root hair genotypes (W90 (Zaoyangmai) and W100 (Shengen)) were selected based on root hair length screening. Among them, W90 is an American variety, while W100, W106, and W136 are Chinese wheat landraces from Fujian, Shanxi, and Henan Province, respectively. Following seed sterilization, germination, and agar medium cultivation, the seed roots were collected on the fourth day of germination and rapidly frozen in liquid nitrogen (three biological replicates for each sample). RNA extraction followed the user manual of the SteadyPure Plant RNA Extraction Kit (Accurate Biology, Qingdao, China). A NanoDrop 2000 (Thermo, Wilmington, NC, USA) was utilized to evaluate the purity and concentration of RNA.

### 4.4. Development of cDNA Library and Transcriptome Sequencing

mRNA enrichment from RNA was performed via utilizing Oligo (dT) beads, followed by random fragmentation into short fragments. These fragments were reverse-transcribed into cDNA and subjected to ligation, A-tailing, and end repair into sequencing junctions. cDNA fragments were selected, and sequencing libraries were generated by amplification with the aid of junction sequences. The quantification of cDNA libraries was firstly conducted by applying Qubit 2.0, with the detection of insert size through Agilent 2100. Accurate quantification of the effective concentration was performed via qPCR to assess the library quality.

The Illumina HiSeq 2500 platform performed 2 × 100 bp double-ended sequencing on qualified cDNA libraries to obtain raw reads. Sequencing data underwent filtration using Fastp [87]: removal of reads with spliced sequences, paired reads with over 10% N, and paired reads with over 50% of low-quality (Q ≤ 20) bases. Clean reads were obtained for subsequent analysis. These clean reads were compared with the existing wheat genome (http://plants.ensembl.org/Triticum_aestivum/Info/Index, accessed on 10 July 2023) and structural annotation files using HISAT2 [88], and the results were subjected to statistical analysis.

### 4.5. Screening and Analyzing Differentially Expressed Genes (DEGs)

Fragments Per Kilobase of transcript per million fragments mapped (FPKM) values was adopted for the quantification of gene expression levels, and the read comparison process employed the featureCounts program [89]. DESeq2 [90] conducted differential expression analyses of the samples. Subsequently, the False Discovery Rate (FDR) was acquired by tuning the probability of hypothesis testing (*p* value) for multiple testing using the Benjamini–Hochberg method [91]. The final list of DEGs was screened based on |log_2_^Fold Change^| ≥ 2 and FDR < 0.05.

### 4.6. Functional Annotation of DEGs

Alignment of all DEGs with the Kyoto Encyclopedia of Genes and Genomes (KEGG), euKaryotic Orthologous Groups (KOG), Swiss-Prot, Gene Ontology (GO), and NCBI nonredundant (NR) databases was conducted by applying the BLAST program (E value < 10^−5^). Through the application of the hypergeometric test [92] based on the KEGG and GO terms database, we discovered a number of significantly enriched GO terms and pathways. Enrichment was measured as the ratio of the DEGs to the genes in the entire genome, within the same GO term or pathway.

### 4.7. MapMan and PPI Analysis

Metabolic and gene regulatory networks for root hair development were established using MapMan v 3.6.0RC1 with shared DEGs in W106/W90, W106/W100, W136/W90, and W136/W100. The submission of the protein sequences of the shared DEGs to the STRING database (https://cn.string-db.org/, accessed on 2 September 2023) with the species parameter set for wheat was conducted. The combined score was set to be greater than 0.6, and interactions between homologous proteins of DEGs were examined and displayed through adopting Cytoscape (v3.7.2).

### 4.8. qRT-PCR Validation

Root RNA samples from long root hair genotypes (W106 and W136) and short root hair genotypes (W90 and W100) were prepared using the agar culture dish method described in Section 2.3. The evaluation of RNA quality was performed using a NanoDrop 2000 spectrophotometer (Thermo Fisher Scientific, Wilmington, NC, USA). The Evo M-MLV Reverse Transcription Kit for qPCR (Accurate Biology, Qingdao, China) was used to obtain the cDNA. A SYBR Green Pro Taq HS Premixed type Kit (Accurate Biology, Qingdao, China) was utilized for the Quantitative fluorescence experiments. PCR was conducted using QuantStudio™ 3 Real-Time PCR Instruments (Applied Biosystems, Thermo Fisher Scientific, Waltham, MA, USA) according to the following protocol: 95 °C for 30 s, followed by 40 cycles of (95 °C for 5 s and 60 °C for 30 s). The dissociation curve was programmed to gradually increase the temperature from 60 °C to 95 °C, with a duration of 5 s per step and an increment of 0.5 °C. The specificity of the primers was confirmed through analysis of the dissolution curves. Actin was chosen as an internal reference for the purpose of normalizing the expression value. The original fluorescence quantitative PCR Ct value was exported, and the −ΔΔCt relative quantification method was employed for calculating the relative gene expression value. The qRT-PCR primers are shown in Supplemental Table S5.

### 4.9. Statistical Analysis

IBM SSPS Statistics 22 conducted the statistical analysis of the data. To determine the significance of the differences, the least significant difference (LSD) test was utilized in a One-way ANOVA, with a *p*-value of less than 0.05. The data significance was determined by applying the Waller–Duncan letter labeling method.

## 5. Conclusions

Root hairs, specialized epidermal cells located in plant roots, have a pivotal function in facilitating the absorption of water and essential mineral nutrients for plants. The ultimate length of these hairs is governed by both plant species and genotype, and is subject to modulation by various environmental factors, such as nutrient and water availability. Understanding the control of root hair length genes could have significant ramifications for the advancement of wheat varieties with enhanced drought resistance and tolerance, as well as improved nutrient uptake capabilities. In order to explore wheat root hair length regulatory genes, we investigated root hair length during the germination of wheat samples of 261 genotypes, screened two long root hair and two short root hair extreme materials, and performed transcriptome sequencing. By analyzing the interactions of DEGs, metabolic pathways, and functions, a number of genes upregulated in long hair length/short root hair genotypes were identified, which were mainly involved in hormone signaling pathways, transcription factor regulation, secondary metabolic pathways, ROS steady state, and other biological functions. It was hypothesized that these genes had a positive contribution to the long root hair phenotypes of W106 and W136. The results offer novel candidate genes for the genetic improvement of wheat root traits, which can be expected to play a role in increasing wheat yield.

## Figures and Tables

**Table 1:** Root transcriptome sequencing data output and genome comparison results of wheat of long root genotypes (W106 and W136) and short root genotypes (W90 and W100).

Sample	Raw Reads	Clean Reads	Reads Mapped	Unique Mapped	Multi Mapped	‘+’ Mapped	‘−’ Mapped	Exon Mapped	Expressed Gene Number
W90-1	65,362,590	59,353,078	53,608,182 (90.32%)	49,153,912 (82.82%)	4,454,270 (7.50%)	24,573,452 (41.40%)	24,580,460 (41.41%)	8,363,394 (91.22%)	88,280
W90-2	61,327,388	58,045,598	53,142,825 (91.55%)	49,217,176 (84.79%)	3,925,649 (6.76%)	24,608,886 (42.40%)	24,608,290 (42.39%)	8,332,918 (91.49%)	88,250
W90-3	73,110,780	68,218,444	62,857,440 (92.14%)	58,143,204 (85.23%)	4,714,236 (6.91%)	29,071,891 (42.62%)	29,071,313 (42.62%)	9,882,370 (91.59%)	89,285
W100-1	63,157,218	57,594,580	51,858,044 (90.04%)	47,556,819 (82.57%)	4,301,225 (7.47%)	23,781,711 (41.29%)	23,775,108 (41.28%)	7,891,593 (91.27%)	88,124
W100-2	55,525,334	46,352,918	43,341,067 (93.50%)	40,140,499 (86.60%)	3,200,568 (6.90%)	20,075,390 (43.31%)	20,065,109 (43.29%)	6,675,681 (92.42%)	85,594
W100-3	63,619,112	59,787,134	54,564,001 (91.26%)	50,472,705 (84.42%)	4,091,296 (6.84%)	25,241,384 (42.22%)	25,231,321 (42.20%)	8,325,128 (91.57%)	89,752
W106-1	67,132,016	62,597,994	58,960,629 (94.19%)	54,803,393 (87.55%)	4,157,236 (6.64%)	27,400,681 (43.77%)	27,402,712 (43.78%)	9,420,269 (92.63%)	87,203
W106-2	78,750,774	73,059,016	68,344,337 (93.55%)	63,408,836 (86.79%)	4,935,501 (6.76%)	31,702,820 (43.39%)	31,706,016 (43.40%)	10,861,159 (92.35%)	88,279
W106-3	71,615,688	65,291,454	61,261,231 (93.83%)	56,812,593 (87.01%)	4,448,638 (6.81%)	28,405,411 (43.51%)	28,407,182 (43.51%)	9,774,520 (92.51%)	86,945
W136-1	96,589,012	90,374,388	83,210,404 (92.07%)	76,814,622 (85.00%)	6,395,782 (7.08%)	38,404,283 (42.49%)	38,410,339 (42.50%)	12,749,176 (91.69%)	88,522
W136-2	103,797,240	97,690,332	90,367,433 (92.50%)	83,612,384 (85.59%)	6,755,049 (6.91%)	41,805,273 (42.79%)	41,807,111 (42.80%)	13,902,102 (91.92%)	88,771
W136-3	92,550,006	87,161,176	81,051,648 (92.99%)	75,268,049 (86.36%)	5,783,599 (6.64%)	37,630,806 (43.17%)	37,637,243 (43.18%)	12,523,229 (92.16%)	88,330

## Data Availability

The data presented in this study are available on request from the corresponding author.

## Supplementary Materials

The following supporting information can be downloaded at https://www.mdpi.com/article/10.3390/ijms25042069/s1.
